# Some Respiratory Symptoms

**Published:** 1888-09

**Authors:** 


### SOME RESPIRATORY SYMPTOMS.

*Puffing of the cheeks at each expiration.*—In reference to your question whether "the puffing of the cheeks at each expiration" be clinical or pathogenetic, I have not had leisure till to-day to refer to the *Reine Arzneimittellehre* of Hahnemann, so as to give you reliable information. You will find on reading *Cinchona*, symptom 361, "Im schlafe erfolgt bald Schnarchendes Einathmen, bald blasendes (bustendes) Ausath men."

This is clearly pathogenetic, and Hahnemann makes himself responsible for its accuracy.

It may further interest you to know, that on February 17th, (1877), I was called to see a lady, turned eighty years of age, who had been passing urine and fæces involuntarily for some days. She suddenly became comatose, in which condition she was at my visit; her limbs were flaccid, so that when raised they fell back to their former position, and remained perfectly useless. She was lying on her back, sliding down in bed, quite unconscious to pinching, but loud calling into her ears aroused some momentary consciousness; no intelligible answer to questions, however, could be elicited. Her face was intensely flushed, and she was bathed in a profuse, hot perspiration; the lips blue-red, respiration labored, loud, and stertorous; but what was most marked among these fatal symptoms was *puffing blowing out of the cheeks at each expiration.* I told the family that, considering the great age of the patient, the case seemed to me to be hopeless. I dissolved five globules of *China* in a tumbler of water; one teaspoonful was given immediately, with directions that the dose should be repeated every twenty minutes. After the second dose there was manifest improvement, and after the third dose the patient opened her eyes and spoke. She made a rapid recovery, and is now in her usual health. The globules used had been moistened with a tincture of the 200th (Lehrman), which had been refilled with spirit many times, so that what the positive dilution may have been I could not tell. The limit, where a remedy loses its power, has yet to be ascertained. By the way, the various translations of this symptom of *Cinchona* are strangely inaccurate, seeing that it really means that "in sleep the inspirations are sometimes snoring, the expirations sometimes puffing."

The most accurate translation I have seen is that of Jourdain's rendering into French, "Pendant le Sommeil, tantôt il ronfle en aspirant, tantôt il souffle en expirant." The only other remedy I am acquainted with, having a similitude to the "puffing expiration," is Plumbum.

The symptom has been extracted from the valuable works of Zanquerel de Planche, under Coma of *Lead.* "He moves his lips as in smoking." I have had no opportunity of verifying this symptom of *Plumbum.**

*Moving up and down of larynx.*—With regard to the "up and down movement of the larynx," I know of no remedy corresponding to it, and have generally found it a sure sign of approaching dissolution; but on two or three occasions during a long experience I have had recoveries under *Lycopodium*, when given at the very beginning of the symptom. I would, however, speak cautiously on this matter, and not hazard anything positive until we have further experience on the point.

In regard to the *fan-like movement of the alœ nasi*, I have, up to this date, found it perfectly reliable as an indication; but I have seen practitioners on the point of losing their patients, as well as their courage, by neglecting to repeat the doses quickly when the urgency of the symptoms demanded frequent doses. Hahne-

*According to Dr. Berridge, Creosote has: "Breath quick and labored, puffing of cheeks and violent working of nostrils." He also states that Chloroform causes puffing of the cheeks when breathing. (*Vide The Organon*, vol. 2, p. 338.)

mann long ago pointed out that, when the symptoms were active, the action of the dose was quickly lost, and required frequent repetition. In the notes to his *Organon*, he tells us that in urgent cases of cholera the dose may be repeated every five minutes. I have repeatedly saved life by keeping these hints in remembrance. Many years ago I recollect Dr. Drury calling on me to say that he had a severe case of measles under his care, where *Lycopodium* failed, although the fan-like movement was present. He kindly allowed me to visit the case with him, and certainly the prospects were very hopeless; the thoracic wheezing was great, the countenance bloated through congestion and imperfect aeration of the blood; the fan-like motion was very rapid. I advised that a dose of *Lycopodium* should be repeated every fifteen minutes (instead of every two hours, as had been the case), and by the afternoon—our visit was in the forenoon—the child was safe and recovered. I should like to interrogate somewhat closely the reported failures where the fan-like motion is distinctly marked. (Extract from a letter of David Wilson, M. D., London, in *The Organon*, vol. 2, p. 339, *et seq.*)

